# Kid-Short Marfan Score (Kid-SMS) Is a Useful Diagnostic Tool for Stratifying the Pre-Test Probability of Marfan Syndrome in Childhood

**DOI:** 10.3390/diseases3010024

**Published:** 2015-03-12

**Authors:** Veronika C. Stark, Florian Arndt, Gesa Harring, Yskert von Kodolitsch, Rainer Kozlik-Feldmann, Goetz C. Mueller, Kristoffer J. Steiner, Thomas S. Mir

**Affiliations:** 1 Clinic for Pediatric Cardiology, University Heart Center, Martinistr. 52, 20251 Hamburg, Germany; E-Mails: ve.stark@uke.de (V.C.S.); f.arndt@uke.de (F.A.); g.harring@uke.de (G.H.); r.kozlik-feldmann@uke.de (R.K.-F.); go.mueller@uke.de (G.C.M.); k.steiner@uke.de (K.J.S.); 2 Clinic for Cardiology, University Heart Center, Martinistr. 52, 20251 Hamburg, Germany; E-Mail: kodolitsch@uke.de

**Keywords:** Marfan syndrome, revised Ghent Criteria, Kid-SMS, diagnosis, childhood, quality of life

## Abstract

Due to age dependent organ manifestation, diagnosis of Marfan syndrome (MFS) is a challenge, especially in childhood. It is important to identify children at risk of MFS as soon as possible to direct those to appropriate treatment but also to avoid stigmatization due to false diagnosis. We published the Kid-Short Marfan Score (Kid-SMS) in 2012 to stratify the pre-test probability of MFS in childhood. Hence we now evaluate the predictive performance of Kid-SMS in a new cohort of children. We prospectively investigated 106 patients who were suspected of having MFS. At baseline, children were examined according to Kid-SMS. At baseline and follow-up visit, diagnosis of MFS was established or rejected using standard current diagnostic criteria according to the revised Ghent Criteria (Ghent-2). At baseline 43 patients were identified with a risk of MFS according to Kid-SMS whereas 21 patients had Ghent-2 diagnosis of MFS. Sensitivity was 100%, specificity 77%, negative predictive value 100% and Likelihood ratio of Kid-SMS 4.3. During follow-up period, three other patients with a stratified risk for MFS were diagnosed according to Ghent-2. We confirm very good predictive performance of Kid-SMS with excellent sensitivity and negative predictive value but restricted specificity. Kid-SMS avoids stigmatization due to diagnosis of MFS and thus restriction to quality of life. Especially outpatient pediatricians and pediatric cardiologists can use it for primary assessment.

## 1. Introduction

Marfan syndrome (MFS) is an inherited connective tissue disorder with multifaceted phenotype involving cardiac, ophthalmologic and skeletal symptoms as well as skin, lung and dura abnormalities [1,2]. Due to age dependent organ manifestation, diagnosis of Marfan syndrome, especially in children, is sophisticated [3,4]. A major aspect in care of patients who are suspected of Marfan syndrome is to assure correct diagnosis of MFS as soon as possible but also to strongly avoid false diagnosis to prevent stigmatization with chronic disease, which can cause various restrictions in quality of life. Decreased quality of life, challenges of education, work and family life, depression and anxiety in patients with diagnosed MFS could be shown before [5]. Therefore, especially in childhood, where organ manifestations may not be fully present, a safe follow-up regime for patients who are suspected of MFS is necessary until definite diagnosis of MFS is possible.

Since 1996, diagnosis of MFS according to Ghent Criteria has been established, including genetic analysis with detection of *FBN1* mutation [6,7]. Loeys *et al.* published revised Ghent Criteria (Ghent-2) (Table 1: Revised Ghent Criteria) in 2010 to improve diagnosis [8]. Ghent-2 is still the gold standard for diagnosis of MFS. It includes organ manifestations of MFS and allows diagnosis in different constellation of clinical symptoms. Although Ghent-2 is a major step forward, its utility in children is still restricted with expensive and technically advanced diagnosis and due to age dependent organ manifestation.

In 2012 we published the Kid-Short Marfan score (Kid-SMS) (Table 2: Kid-Short Marfan Score) to stratify the pre-test probability of MFS in early childhood, especially upon first presentation of patients who are suspected of MFS [9]. Kid-SMS allows early risk stratification without stigmatization of children with chronic disease too early, but enables a safe follow-up regime and flexibility in stratification class and diagnosis, which is indispensable concerning age dependent onset of organ manifestations in MFS.

Due to the life limiting aspect of cardiac pathologies in MFS, and the possibility of early medical treatment to avoid surgery or even life-threatening events like dissection or aortic rupture, Kid-SMS concentrates on those symptoms [10,11]. In addition we included ectopia lentis, skeletal features and family history in the score. According to resulting pre-test probability class Kid-SMS recommends further diagnostic steps and follow-up strategies. This study now re-evaluates the predictive performance of Kid-SMS for MFS. We also want to estimate the advantage and disadvantage of using Kid-SMS in clinical life. Finally we review its usefulness and importance in the diagnosis of MFS in childhood.

**Table 1:** Revised Ghent Criteria (Ghent-2) for diagnosis of MFS [7].

Dilatation or Dissection of Aorta	
Ectopia Lentis	
**Systemic Involvement (Positive if at Least 7/20 Points)**	**Score**
Pectus carinatum	2
Pectus excavatum or chest asymmetry	1
Reduced upper segment/lower segment AND increased armspan/height AND no scoliosis	1
Characteristic face (3 of 5 facial features—dolichocephaly, enophthalmus, downslanting palpebral fissures, malar hypoplasia, retrognathia)	1
Wrist AND thumb sign	3
Wrist OR thumb sign	1
Scoliosis or thoracolumbar kyphosis	1
Reduced elbow extension (<170°)	1
Plain pes planus	1
Hindfoot deformity	2
Protusio acetabulae	2
Myopia (>3diopters)	1
Mitral valve prolaps	1
Spontaneous pneumothorax	2
Striae atrophicae	1
Lumbosacral dural ectasia	2
***FBN1* mutation**	
Confirmed MFS:	
-Dilatation/Dissection of aorta + ectopia lentis OR systemic manifestation OR *FBN1* mutation	
-Family history of MFS + Dilatation/Dissection of aorta OR ectopia lentis OR systemic involvement	

MFS, Marfan syndrome.

**Table 2:** Kid-Short Marfan Score (Kid-SMS) [8].

Required Manifestations	Risk Category for Likelihood of MFS
SV + EL	**Very high risk** (Diagnosis of MFS according Ghent-2)
SV + MVP + TVP SV + PA SV + 3 Skeletal Features	**High risk** (Patient is at high risk of MFS. Complete examination of all symptoms of the revised Ghent Criteria is strictly recommended as soon as possible. Patient should see MFS specialists)
EL + MVP + TVP EL + PA	
Family history SV	**Moderate risk** (Patient needs to be verified or excluded with further diagnostic procedures other than or echocardiography and clinical examination)

SV, dilatation of sinus of valsalvae; EL, ectopia lentis; MVP, mitral valve prolapse; TVP, tricuspid valve prolapse; PA, dilatation of pulmonary artery; 3 skeletal features, at least 3 skeletal features of the systemic score of the revised Ghent Criteria; Ghent-2, revised Ghent Criteria; MFS, Marfan syndrome.

## 2. Experimental Section

Between January 2012 and August 2014 we prospectively investigated 106 pediatric patients who were suspected of having MFS (39 female, 67 male) in the Marfan clinic at the University Heart Center Hamburg. All patients had complete assessment of manifestations of MFS with echocardiography and clinical examination. If necessary and reasonable we also accomplished magnetic resonance imaging (MRI) and genetic analysis.

At baseline, children were examined according to Kid-SMS. At baseline and follow-up visit, diagnosis of MFS was established or rejected using current standard diagnostic criteria according to Ghent-2.

We performed echocardiography with General Electric Vivid 7 with 10, 5 and 3 MHz probes. To obtain measurement of aortic root diameters we operated in parasternal long axis view on 2D and M-Mode images using leading edge to leading edge technique at end diastole. We evaluated dilatation of sinus of valsalvae according to *Roman et al.* [12]. For the measurement of the pulmonary artery, we operated in parasternal short axis view and evaluated using Z-score [13]. We estimated mitral valve prolapse and tricuspid valve prolapse in four-chamber view and parasternal long axis. An experienced pediatric cardiologist performed the examination.

We collected data using Filemaker software V.10 pro advanced. To perform statistical analysis, we used SPSS V16.0 and for tables and figures we used Microsoft Excel 2003 and SPSS V16.0.

We expressed quantitative variables as means with standard deviation and qualitative data as numbers. For comparison of quantitative data between groups we used unpaired t test and considered *p* values < 0.05 as significant. We compared qualitative data by Fisher’s exact test. We also determined positive likelihood ratio for Kid-SMS. Likelihood ratio larger than ten was considered as extremely reliable to identify patients with MFS, whereas values between three and ten were useful and values below three were not useful. Finally, we used Kaplan Meier analysis and log-rank test to display age of diagnosis with Kid-SMS and Ghent-2 [14]. Age of diagnosis of MFS concerning Ghent-2 and age of risk stratification with Kid-SMS were defined as endpoint of surveillance. Kid-SMS was considered as positive as soon as moderate risk for MFS was stratified. Negative Kid-SMS was considered as negative test. Again we considered *p* values < 0.05 as significant.

To assess clinical data and samples, we obtained informed consent of patients or parents of patients. The study was approved by the Hamburg ethical board (Project identification code: PV 4005).

## 3. Results and Discussion

### 3.1. Results

**Figure 1:**
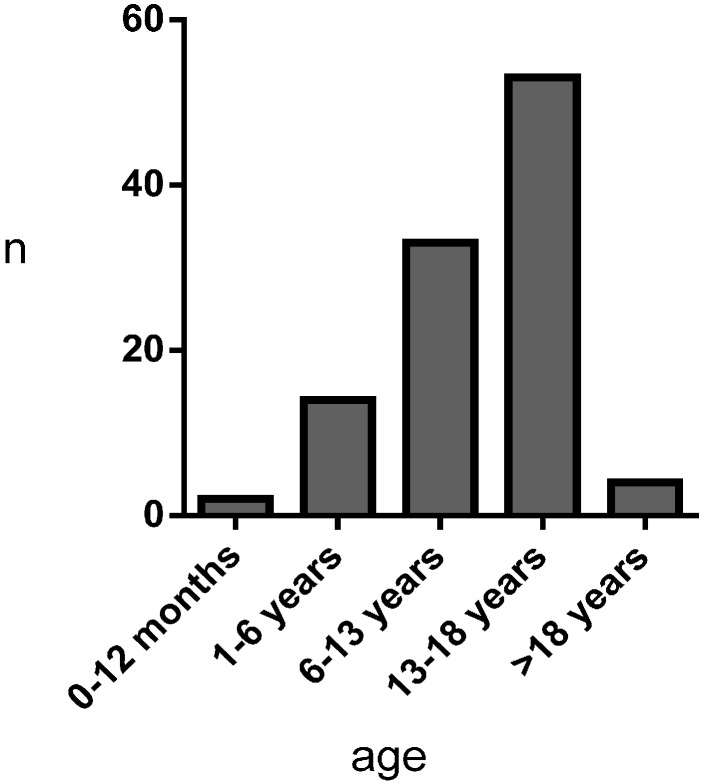
Age at first presentation to Marfan clinic.

Mean age of first presentation to Marfan clinic was 12.18 ± 5.49 years (2 patients 1–12 months, 14 patients 1.01–6.00 years, 33 patients 6.01–13.00 years, 53 patients 13.01–18.00 years, 4 patients > 18 years) (Figure 1).

At baseline, Kid-SMS stratified 43 children at risk of having MFS; 20 of those patients also had Ghent-2 diagnosis of MFS (Table 3). During follow-up period, four patients were diagnosed according to Ghent-2, whereas Kid-SMS stratified a risk of MFS in those patients at baseline. Another five patients showed *FBN1* mutation without Ghent-2 diagnosis of MFS, whereas Kid-SMS identified two at a risk of MFS. Besides, 14 patients were identified at risk of MFS without current diagnosis according to Ghent-2 or *FBN1* mutation (Table 4).

**Table 3:** Fourfold table of risk stratification with Kid-SMS and diagnosis with Ghent-2 at baseline (first presentation). (Ghent-2 Pos, revised Ghent Criteria positive; Ghent-2 Neg, revised Ghent Criteria negative; Kid-SMS Pos, Kid-Short Marfan Score positive; Kid-SMS Neg, Kid-Short Marfan Score negative).

*n* = 106	Ghent-2 Pos	Ghent-2 Neg
Kid-SMS Pos	21	22
Kid-SMS Neg	0	63

**Table 4:** Fourfold table of risk stratification with Kid-SMS and diagnosis with Ghent-2 at follow-up visit, *p* < 0.05. (Ghent-2 Pos, revised Ghent Criteria positive; Ghent-2 Neg, revised Ghent Criteria negative; Kid-SMS Pos, Kid-Short Marfan Score positive, Kid-SMS Neg, Kid-Short Marfan Score negative).

*n* = 106	Ghent-2 Pos	Ghent-2 Neg
Kid-SMS Pos	24	19
Kid-SMS Neg	0	63

Sensitivity of Kid-SMS was 100% (CI 95% 0.86 to 1.00), whereas specificity was 77% (CI 95% 0.66 to 0.85). Positive predictive value was 56% (CI 95% 0.40 to 0.71) and negative predictive value 100% (CI 95% 0.94 to 1.00). Likelihood ratio was 4.3, which reveals good predictive performance of Kid-SMS. Fisher’s exact test showed statistic significance (*p* value < 0.05).

At follow-up visit, detailed analysis of risk stratification at baseline with Kid-SMS in comparison with diagnosis of MFS according to Ghent-2 showed “very high risk” in two patients with MFS and none in patients without diagnosis according to Ghent-2. “High risk” was graded in 20 patients with diagnosis according to Ghent-2 and two patients without diagnosis according to Ghent-2 or *FBN1* mutation. “Moderate risk” was graded in five patients with MFS, two with *FBN1* mutation, and 12 patients without MFS (Table 5).

**Table 5:** Comparison of risk stratification with Kid-SMS and diagnosis according to Ghent-2 at follow-up visit. (Ghent-2 Pos, revised Ghent Criteria positive; Ghent-2 Neg, revised Ghent Criteria negative; *FBN1 Pos*, *FBN1* mutation positive; *FBN1 Neg*, *FBN1* mutation negative; SV, dilatation of sinus of valsalvae; PA, dilatation of pulmonary artery; MVP, mitral valve prolapse; TVP, tricuspid valve prolapse; 3Skel, at least 3 skeletal features of the systemic score of the revised Ghent Criteria; EL, ectopia lentis; FH, family history).

	Patients Ghent-2 Pos (Ghent-2), *n* = 27	Patients Ghent-2 Neg, *n* = 79	
		Patients *FBN1 Pos*, *n* = 5	Patients *FBN1 Neg*, *n* = 74
**Very high risk SV + EL**	2	0	0
**High risk SV + 3Skel**	8	0	2
**High risk SV + MVP + TVP**	7	0	0
**High risk SV + PA**	3	0	0
**High risk EL + MVP + TVP**	0	0	0
**High risk EL + PA**	2	0	0
**Moderate risk (FH)**	2	2	7
**Moderate risk (SV)**	3	0	5
**Negative**	0	3	60

**Figure 2:**
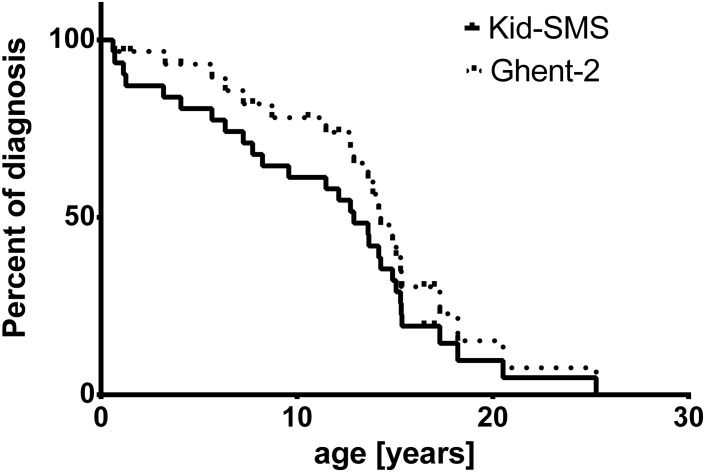
Kaplan-Meier analysis of age of diagnosis with Ghent-2 (Revised Ghent Criteria, dashed line) and risk stratification of MFS (very high risk, high risk, moderate risk) with Kid-SMS (Kid-Short Marfan Score, continuous line), *p* = 0.2, ns.

Mean age of risk stratification with Kid-SMS was 10.63 ± 1.23 years, whereas age of diagnosis with Ghent-2 was 12.07 ± 1.16 years. In Kaplan-Meier analysis the endpoint of presented curves is age of diagnosis of MFS concerning Ghent-2 (dashed line) and age of declared risk stratification with Kid-SMS, which may be very high risk, high risk or moderate risk (continuous line). Analysis did not show significant difference concerning age of diagnosis or risk stratification (*p* = 0.2, ns) (Figure 2).

### 3.2. Discussion

In this study, all patients with diagnosed MFS according to Ghent-2 were stratified for risk of MFS with Kid-SMS, which is shown by perfect negative predictive value. Kid-SMS also defines a group where diagnosis cannot be assured yet but Kid-SMS recommends a safe follow-up regime. This avoids stigmatization and unnecessary restrictions in quality of life due to false diagnosis of chronic disease. Especially children should be protected from stigmatization to support psychosocial development und encourage setting of resources for psychosocial coping in life.

At first presentation (baseline), Kid-SMS identified a distinctly larger group of patients with a risk of MFS than patients with diagnosis according to Ghent-2. During follow-up period, three patients were diagnosed according to Ghent-2, whereas Kid-SMS already stratified a risk of MFS in those patients at baseline. Nevertheless, there is a group of children with stratified risk of MFS where Ghent-2 was negative at final visit. Some of those (2) showed *FBN1* mutation without diagnosis with Ghent-2. Others (14) were neither diagnosed with Ghent-2 nor showed *FBN1* mutation.

Both patients with *FBN1* mutation were at a moderate risk of MFS due to positive family history of MFS. Those patients were aged 1.1 and 8.4 years at final presentation. Due to age dependent organ manifestation in MFS symptoms may not be present especially in early childhood. Those patients need a strict follow-up regime to evaluate upcoming manifestations of MFS later on. They should be treated as patients with MFS and may subsequently be diagnosed according to Ghent-2.

In three patients with *FBN1* mutation, Kid-SMS did not predict a risk of MFS. One showed *FBN1* mutation with isolated mitral and tricuspid valve prolapse. Thus, this patient would of course be followed up by cardiological evaluation. Consequently, in case of aortic enlargement or other cardiac pathologies, further diagnostic tools and necessary treatment would be induced. Both of the others showed a *FBN1* mutation with a positive systemic score and no vascular involvement aged 15.8 and 17.6 years. Those children had no vascular symptoms, which are life-limiting in MFS. Even though MFS is unlikely in those patients they may have another connective-tissue disorders like MASS phenotype associated with *FBN1* mutation.

Prediction of risk of MFS with Kid-SMS in patients without diagnosis according to Ghent-2 or genetic analysis was established in two patients who showed dilatation of sinus of valsalvae in combination with skeletal symptoms.

Another five patients were at moderate risk of MFS because of dilatation of sinus of valsalvae. Even though there were no other symptoms of MFS those patients will be followed up in a specialized center and may be diagnosed later on with another disease involving thoracic aortic aneurysm [15]. Seven showed moderate risk of MFS because of family history. After additional examinations like genetic analysis and MRI, MFS was very unlikely.

Although Kid-SMS also predicts a risk of MFS in some patients without disease, it will probably cover patients with other syndromes, including thoracic aortic aneurysms like Loeys-Dietz syndrome or Shprintzen-Goldberg syndrome, that also need regular follow-up. The main criteria in Kid-SMS are cardiac and vascular involvement of MFS. Dilatation of sinus of valsalvae is the most common feature in children with MFS [16]. And it is the symptom that is most important for medical and surgical treatment. Therapies with beta-blocker as well as with AT-1 antagonists avoid progression of aortic root dilatation, surgery and, in the end, life-threatening events [17,18]. Because of interference with other connective tissue diseases where those vascular symptoms occur, differentiation with Kid-SMS is difficult and specificity is restricted. To at least optimize power of discrimination dilatation of sinus of valsalvae is not included in the score as an isolated symptom but in combination with other features, which are typical for MFS like dilatation of pulmonary artery and skeletal features.

We were not able to examine larger groups of children with other syndromes, including thoracic aortic aneurysms, to analyze exact power of discrimination of Kid-SMS. Although improvement of differential diagnosis was one reason to revise the Ghent criteria, this is important for Kid-SMS but not the main focus [8]. Consideration of differential diagnosis should attend patients in follow-up regimes until definite diagnosis is assured.

In total, at risk stratification for MFS with Kid-SMS, children were younger at diagnosis than according to Ghent-2. Early identification of patients with a risk of MFS enables their inclusion in a safe follow-up regime and can identify the possible need for prophylactic medical treatment. Indeed, first presentation to a Marfan clinic is not the time of incidence of symptoms and thus not the time of detection of Kid-SMS, which may be the reason for absent significance. To evaluate a better reliable age of pre-test probability with Kid-SMS use of the score in outpatient children with reasonable suspicion of MFS is necessary. In our experience we see many children where Kid-SMS is the first diagnostic tool that correctly supports or excludes diagnosis of MFS.

According to level of risk stratification with Kid-SMS, further examinations are recommended. Patients with very high risk of MFS, which is equivalent to diagnosis according Ghent-2, must be presented to a specialized center of MFS. In patients with a high risk or moderate risk with dilatation of SV, full examination concerning Ghent-2 has to be completed urgently, even though risk of MFS is lower in moderate risk patients. In areas where access to MRI and genetic analysis is limited, further examinations can be delayed as long as regular echocardiographic follow-up is assured. Also, performance of MRI may not be possible sometimes due to low age of patients where quality of MRI data is reduced due to fast heart rate and children would need anesthesia for examination. Patients with moderate risk of MFS due to positive family history further examinations are recommended to include or exclude them in a follow-up regime. Due to age dependent onset of organ manifestation, many children of our study group changed level of risk stratification during follow-up, which represents flexibility of the score. Except the change from negative to positive risk stratification, we only comment on first level of risk stratification of MFS in results and discussion. A restriction of the application of Kid-SMS, especially in outpatient care, is the performance of echocardiography, which requires expert experience. Nevertheless, this is much more easily available than a specialized pediatric center for MFS.

Unfortunately, this study does not represent the use of Kid-SMS in outpatient children, which is indeed one of the most important skills of the score. To evaluate use of Kid-SMS in those children, further analysis and studies are necessary. But even though our clinic is a specialized center, we used Kid-SMS to give parents first advice for probability of MFS and to decide how urgent further investigations were. Kid-SMS involves symptoms that are also examined to assure diagnosis according to Ghent-2. Thus, performance of the score is not time-consuming and does not require additional examinations, which would limit its usefulness, especially in outpatient care. In summary, practice of Kid-SMS in this patient group was valuable for children with assumed MFS. Kid-SMS showed very good sensitivity and restricted specificity. Likelihood ratio shows good predictive performance of stratifying risk of MFS with Kid-SMS. It was easily executable and not time-consuming. After all, Kid-SMS is not supposed to replace Ghent-2 diagnosis of MFS. Instead, it should be used in addition to Ghent-2 to improve diagnosis in childhood and assess early pre-test probability of MFS. Patients without a definite diagnosis or exclusion of disease are thus not stigmatized with the diagnosis of MFS but in a safe follow-up regime.

Especially pediatricians and pediatric cardiologists not working in specialized centers can use it for a risk assessment and to estimate whether or not a patient requires transfer to a specialized center. Particularly for them, Kid-SMS is an easily executable diagnostic tool for first screening of MFS in childhood.

## 4. Conclusions

Whereas diagnosis of MFS is sophisticated, Kid-SMS is a useful tool to assess early pre-test probability of MFS in childhood, especially for pediatricians and pediatric cardiologists in out-patient care.
